# Delayed-type heparin allergy: diagnostic procedures and treatment alternatives--a case series including 15 patients

**DOI:** 10.1097/WOX.0b013e31818def58

**Published:** 2008-12-15

**Authors:** Claudia Pföhler, Cornelia SL Müller, Gerhard Pindur, Hermann Eichler, Hans-Joachim Schäfers, Ulrich Grundmann, Wolfgang Tilgen

**Affiliations:** 1 Saarland University Hospital, Department of Dermatology, Homburg/Saar, Germany; 2 Saarland University Hospital, Institute of Clinical Hemostaseology and Transfusion Medicine, Homburg/Saar, Germany; 3 Saarland University Hospital, Department of Thoracic and Cardiovascular Surgery, Homburg/Saar, Germany; 4 Saarland University Hospital, Department of Anaesthesiology and Intensive Care, Homburg/Saar, Germany

**Keywords:** heparin, allergy, alternatives, pregnancy, cardiac surgery, thrombosis

## Abstract

Delayed-type hypersensitivity reactions (DTHRs) after subcutaneous application of unfractionated heparins or low-molecular-weight heparins are not uncommon. Standard allergological testing usually includes intracutaneous skin tests and patch testing of different heparins, heparinoids, and thrombin inhibitors followed by subcutaneous and/or intravenous challenge with skin test-negative drugs. We present data from a single-center case series of 15 patients with DTHR after low-molecular-weight heparin administration. Intracutaneous testing that can be considered as gold standard identified the suspicious elicitor in 11 (73.4%) of 15 of the patients. Patch testing was positive in 5 (33.4%) of 15 of the patients and was only positive in patients who were also reacting in the intradermal testing. Intravenous challenge with heparin sodium was performed in 10 of 15 patients and was well tolerated in all cases, despite prior positive intracutaneous tests with the same substance. Intracutaneous documentation of DTHR was not an adequate predictor of intravenous challenge.

## 

Heparins have been used for the treatment and prophylaxis of thromboembolic diseases for about 6 decades [1]. All heparins are sulfated mucopolysaccharides with a highly negative charge. Unfractionated heparins (UFHs), such as heparin calcium and heparin sodium with a molecular weight of 12 to 20 kd, have to be distinguished from low-molecular-weight heparins (LMWHs) such as enoxaparin, dalteparin, nadroparin, and certoparin with a molecular weight of 4 to 6 kd. Unfractionated heparins are extracted from porcine intestinal mucosa or bovine lung. Low-molecular-weight heparins are manufactured by fractionation of UFH [2]. In addition to heparins, further anticoagulatory drugs are available: semisynthetic heparinoids such as danaparoid sodium, synthetic pentasaccharides such as fondaparinux natrium, and direct thrombin inhibitors, that is, the hirudins lepirudin and desirudin or synthetic thrombin inhibitors such as argatroban and bivalirudin.

Hypersensitivity reactions against heparins, heparinoids, and hirudins are well known and can induce different hypersensitivity reactions according to the classification by Coombs and Gell (Table 1) [3]. Immediate-type reactions (type I reactions), that is, generalized urticaria, angioedema, bronchospasm, and severe anaphylaxis are rare and have been reported for UFH, LMWH, and lepirudin. A severe adverse event of heparins is heparin-induced thrombocytopenia (HIT) type II, a classic type II reaction induced by polyclonal antibodies, usually against the heparin-platelet factor 4 complex [4]. Cutaneous manifestations of HIT type II may include erythemas and skin and mucosal necrosis. The Arthus reaction represents a type III reaction resulting from antigen-antibody complexes and is characterized by inflammation, erythematous induration, and edema at the injection site, which can result in subsequent hemorrhage and necrosis [5]. The most common type of heparin hypersensitivity is the delayed-type hypersensitivity reaction (DTHR), a type IV allergic reaction characterized by itchy eczema and plaques at the injection sites (Figure 1). Histological investigation of skin biopsies from DTHR lesions usually shows a mixed perivascular infiltrate with many eosinophils and dermal edema (Figure 2). These reactions have first been described by Plancherel [6] in 1953 and become evident in nonsensitized individuals within 10 to more than 20 days after treatment initiation. Once sensitized, patients react commonly within 2 to 3 days after reexposure. Until now, the pathomechanism of these DTHR is not completely understood. The heparin molecule itself does not seem to be immunogenic. It is assumed that binding of the molecule to hitherto unknown cutaneous or subcutaneous proteins transfers the hapten heparin into a full antigen [2].

**Table 1:** Overview of Different Anticoagulatory Drugs, Their Way of Application and Testing, Cross-Reactivity With Other Substances and Documented Clinical Features

Anticoagulant	Substance Class	Way of Application	Way of Testing	Cross-Reactivity With	Documented Clinical Features
Heparin calcium	UFH (sulfated mucopolysaccharide)	IV or SC	Undiluted	LMWH, heparin sodium	Skin necrosis, urticaria, bronchospasm, anaphylaxis, HIT type II, DTHR
Heparin sodium	UFH (sulfated mucopolysaccharide)	IV or SC	Undiluted	LMWH, heparin calcium	Skin necrosis, urticaria, bronchospasm, anaphylaxis, HIT type II, DTHR
Dalteparin	LMWH	SC	Undiluted	UFH, other LMWH	Skin necrosis, urticaria, bronchospasm, anaphylaxis, HIT type II, DTHR
Nadroparin	LMWH	SC	Undiluted	UFM, other LMWH	Skin necrosis, urticaria, bronchospasm, anaphylaxis, HIT type II, DTHR
Enoxaparin	LMWH	SC	Undiluted	UFM, other LMWH	Skin necrosis, urticaria, bronchospasm, anaphylaxis, HIT type II, DTHR
Repivarin	LMWH	SC	Undiluted	UFM, other LMWH	Skin necrosis, urticaria, bronchospasm, anaphylaxis, HIT type II, DTHR
Tinzaparin	LMWH	SC	Undiluted	UFM, other LMWH	Skin necrosis, urticaria, bronchospasm, anaphylaxis, HIT type II, DTHR
Certoparin	LMWH	SC	Undiluted	UFM, other LMWH	Skin necrosis, urticaria, bronchospasm, anaphylaxis, HIT type II, DTHR
Pentosan polysulfate	Semisynthetic heparinoid	SC	Undiluted	UFM, LMWH	Skin necrosis, urticaria, bronchospasm, anaphylaxis, fever, chills, HIT type II, DTHR
Danaparoid	Semisynthetic heparinoid	IV or SC	Undiluted	LMWH	Rash, maculopapular exanthemas, pustulosis, pruritus, DTHR
Lepirudin	Recombinant hirudin	IV	Undiluted	Bivalirudin	Anaphylactic and anaphylactoid reactions (including fatal outcome), DTHR, exanthemas, fever, chills
Fondaparinux	Synthetic pentasaccharide	SC (IV possible)	Undiluted	UFM, LMWH	Urticaria, edema, anaphylaxis, rash, DTHR
Bivalirudin	Synthetic thrombin inhibitor	IV	Undiluted	Not reported	Urticaria, anaphylaxis (including fatal outcome)
Argatroban	Synthetic thrombin inhibitor	IV	Undiluted	Not reported	Rash, urticaria, bullous dermatitis, edema

IV indicates intravenous; SC, subcutaneous.

**Figure 1:**
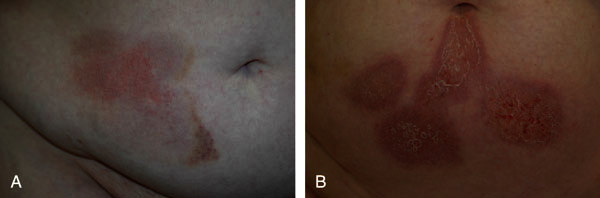
**Erythematous and scaly plaques with inflammatory infiltration as a sign of DTHR in 2 patients after injection of nadroparin (A) or enoxaparin (B) into the skin of the abdominal wall**.

**Figure 2:**
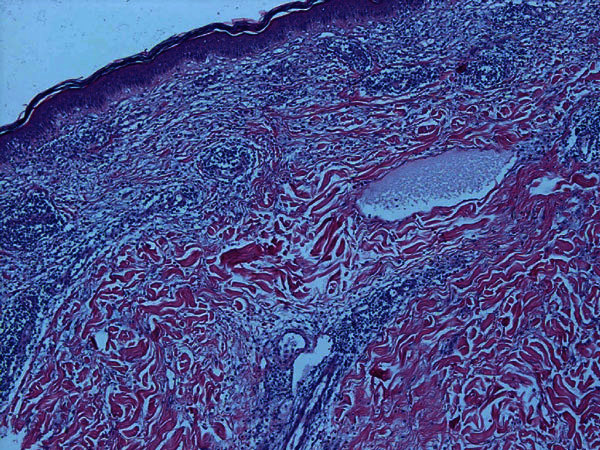
**Focally spongiotic epidermis with mixed in-depth perivascular infiltrate with many eosinophils and dermal edema (hematoxylin and eosin [HE] staining, magnification ×400)**.

Risk factors for the development of DTHR to heparins are female sex, older age, pregnancy, and probably obesity and prolonged exposure to heparins [5,7]. It is still controversially discussed whether the molecular weight of the different heparins also influences the risk for development of DTHR [7-9].

As the clinical picture does not necessarily allow the classification of the underlying allergologic mechanism, allergy testing includes different procedures: skin prick testing, intracutaneous and patch testing of UFH and LMWH, heparinoids, hirudins, and further synthetic thrombin inhibitors, followed by subcutaneous or intravenous challenge. In cases of DTHR, an alternative drug for subcutaneous application can easily be identified using these tests. As several heparins, hirudins, and synthetic thrombin inhibitors are contraindicated during pregnancy, the choice for an alternative drug in pregnant women is more difficult. This is also true for patients undergoing cardiac surgery, in particular, with extracorporeal circulation. In that situation, there is sometimes not enough time to carry out the complete allergologic test procedure if surgery is required without delay. In addition, the alternative substances may have relevant drawbacks in conjunction with extracorporeal circulation. Furthermore, it is still unclear if patients with DTHR have an increased risk to develop systemic complications or HIT type II.

To define possible cross reactions between subcutaneously and intravenously applied heparin, we studied 15 patients with a history of DTHR after heparin application.

## Materials and methods

### Patients

Fifteen patients (3 male, 12 female) with a median age of 49 years (range, 26-72 years) suspected for heparin allergy were examined and tested during a period of 18 months from January 2007 to June 2008 at the allergologic outpatient unit of the Department of Dermatology. Fourteen patients had a history of DTHR after administration of LMWH, 1 patient reported the development of eczema after local application of a heparin ointment. None of the patients had a history of HIT type II. Four female patients had developed itchy plaques on the injection sites of LMWH during pregnancy, and 6 patients required urgent cardiac surgery with extracorporeal circulation for treatment of coronary heart disease or cardiac valve dysfunction.

The patient characteristics are shown in Table 2.

**Table 2:** Characteristics of Patients

Patient	Sex	Age, y	Elicitor	Special Features
1	Female	66	Tinzaparin, certoparin, danaparoid	Heart surgery
2	Female	72	Certoparin	Heart surgery
3	Male	47	Nadroparin	Heart surgery
4	Female	49	Enoxaparin	--
5	Female	55	Enoxaparin, nadroparin	--
6	Female	71	Enoxaparin	Heart surgery
7	Male	65	Heparin ointment	Heart surgery
8	Female	39	Enoxaparin	Pregnancy
9	Female	54	Enoxaparin	--
10	Female	36	Enoxaparin	Pregnancy
11	Male	46	Enoxaparin	--
12	Female	59	LMWH	Heart surgery
13	Female	46	Enoxaparin, certoparin	--
14	Female	36	Enoxaparin, dalteparin	Pregnancy
15	Female	26	Dalteparin, nadroparin	Pregnancy

### Skin allergy tests

**Figure 3:**
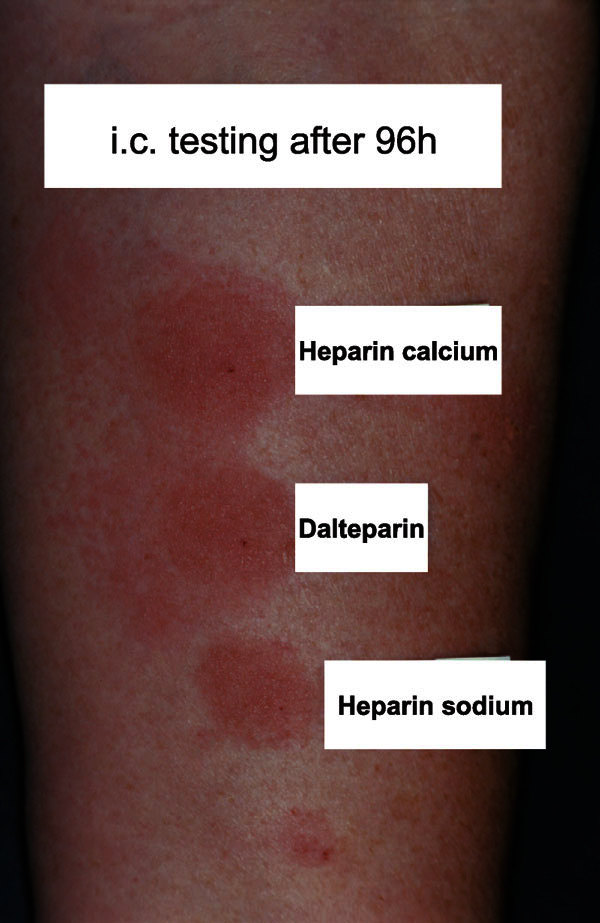
**Results of intracutaneous heparin testing, 96 hours after injection of UFH and LMWH into the volar aspect of the forearm**. The injection of heparin calcium, dalteparin, and heparin sodium led to infiltrated lesions with blisters.

Intracutaneous tests on the inner forearm of the patients included heparin calcium, heparin sodium, dalteparin, nadroparin, enoxaparin, repivarin, tinzaparin, certoparin, pentosan polysulfate, danaparoid, lepirudin, and fondaparinux sodium and were performed using 0.05 mL of the original undiluted drug. The test results were read 20 minutes and 24, 48, 72, and 96 hours after injection (Figure 3). Ten of 15 patients were tested with the whole series of mentioned heparins.

Patch tests with the same drugs were carried out on the back. Patch testing was performed by using the undiluted drug on Curatest chambers (Lohmann und Rauscher, Vienna, Austria). Application was performed for 24 hours. In addition, patch testing included the German standard test series (Hermal, Reinbek, Germany) as well as the preservatives chlorocresol, benzalkonium chloride, and thiomersal that are sometimes used in different brands of heparins. Readings were taken on days 2, 3, and 4 according to International Contact Dermatitis Research Group guidelines.

### Subcutaneous challenge

A subcutaneous challenge was performed by injection of a therapeutic dose of selected skin test-negative drugs into the abdominal skin. Results were obtained after 20 minutes and 24, 48, 72, and 96 hours after drug administration, in some cases, even after 96 and 120 hours.

### Intravenous challenge

Intravenous challenge was performed in 10 of 15 patients reactive to UFH in skin testing and in patients requiring cardiac surgery immediately or with known cardiovascular disease (patients 1, 2, 3, 4, 5, 6, 7, 9, 12, and 13). For intravenous challenge, the patients were hospitalized. We applied heparin sodium as a bolus in a dose of 2500 IU on day 1, 5000 IU as a bolus followed by 7500 IU for 6 hours on day 2, with a follow-up period of 24 hours on an inpatient basis, and a further 24 and 48 hours on an outpatient basis.

## Results

In 11 (73.4%) of 15 patients, the suspected LMWH could be proven as elicitor by intracutaneous testing (Figure 3). In 1 case (patient 3), no LMWHs were tested as heart surgery had to be performed without delay. In a female patient (patient 15) with reported DTHR after application of dalteparin and nadroparin during pregnancy, intracutaneous testing performed after birth of the child was negative. In the case of the patient with a history of eczema after application of a heparin ointment (patient 7), intracutaneous testing showed no pathological reaction. Complete test results of intracutaneous testing are shown in Table 3.

**Table 3:** Results of Intracutaneous Testing

**No**.	Elicitor	Heparin Calcium (Calciparin)	Heparin Sodium (Na-Heparin Braun)	Dalteparin (Fragmin)	Nadroparin (Fraxiparin)	Enoxaparin (Clexane)	Repivarin (Clivarin)	Tinzaparin (Innohep)	Certoparin (Embolex)	Pentosan polysulfate (Fibrezym)	Danaparoid (Orgaran)	Lepirudin (Refludan)	Fondaparinux Sodium (Arixtra)
1	Tinzaparin, certoparin, danaparoid	+	+	+	+	+	+	-	+	+	+	-	-
2	Certoparin	-	-	-	-	-	-	-	+	-	-	-	-
3	Nadroparin	+	+	ND	ND	ND	ND	ND	ND	ND	ND	-	ND
4	Enoxaparin	-	+	-	-	-	-	-	-	-	-	-	-
5	Enoxaparin, nadroparin	+	+	-	+	+	+	+	+	-	+	-	-
6	Enoxaparin	+	+	-	+	+	-	+	-	+	+	+	-
7	Heparin ointment	-	-	-	-	-	-	-	-	-	-	-	-
8	Enoxaparin	ND	ND	ND	+	+	ND	ND	ND	ND	-	ND	-
9	Enoxaparin	+	+	-	-	+	-	+	-	+	-	-	-
10	Enoxaparin	ND	ND	ND	ND	+	ND	ND	ND	ND	-	ND	-
11	Enoxaparin	+	+	+	+	+	+	-	-	-	-	-	-
12	LMWH	ND	+	ND	ND	+	ND	ND	ND	ND	+	-	-
13	Enoxaparin, certoparin	+	+	+	+	+	+	+	+	+	+	-	-
14	Enoxaparin, dalteparin	ND	ND	-	+	+	ND	-	ND	ND	+	ND	-
15	Dalteparin, nadroparin	-	-	-	-	-	-	-	-	-	-	-	-

Results of intracutaneous testing with different drugs: +, positive reaction; -, negative reaction.

ND indicates not done.

Patch testing of suspected drugs was positive in 5 (33.4%) of 15 of the patients. In cases with positive patch tests, patch test results only proved the results of intracutaneous testing. There was no case with a positive result in patch testing without any positive intracutaneous test. Patch testing of the preservatives chlorocresol, benzalkonium chloride, and thiomersal was negative in all patients.

Subcutaneous challenge was performed with different drugs according to the medical history of the individual patient. In 6 patients with negative standard testing (intracutaneous and patch testing), confirmation of an allergic response to heparin was found after subcutaneous challenge. Application of danaparoid and lepirudin was followed by DTHR in 2 patients with negative skin testing and a history of DTHR after enoxaparin administration. Fondaparinux sodium was given subcutaneously in 14 of 15 patients and was well tolerated without development of DTHR in all cases.

Intravenous provocation with heparin sodium was performed as previously described in 10 of 15 patients and tolerated well in all cases.

## Discussion

Adverse reactions to heparin are relatively common. In most cases, patients develop DTHR against LMWH. If patients receive LMWH as thromboembolic prophylaxis after an operation, as a treatment of deep venous thrombosis or as a prophylaxis because of increased risk of thromboembolic diseases, there are many alternatives that should be taken into consideration. Heparinoids such as pentosan polysulfate or danaparoid or recombinant hirudins such as lepirudin are recommended as drugs of first choice because the replacement of 1 LMWH with another LMWH is not appropriate because of cross-reactivity between all LWMHs [7].

In pregnant women, the choice of treatment alternatives is limited because hirudins are contraindicated during pregnancy. Although danaparoid seems to be a good alternative drug for pregnant women at risk of thromboembolic complications, there are several reports indicating that patients experiencing a DTHR against LMWH in the past also developed DTHR after changing to danaparoid administration [10,11]. We could confirm this phenomenon in our collective: 2 of 4 female patients who developed DTHR during pregnancy came to us with DTHR against danaparoid that had been given after development of DTHR after LMWH administration. Another pregnant female patient was challenged subcutaneously with danaparoid after negative intracutaneous and patch testing for this substance and developed DTHR against danaparoid 3 days later. In the fourth female, we did not inject danaparoid subcutaneously.

In the last years, the synthetic pentasaccharide fondapar-inux sodium has become available as an alternative anticoagulant, a drug with only rarely reported cross-reactivity with heparins that may also be applied during pregnancy [12-16]. In our study, 14 of 15 patients had a subcutaneous challenge with fondaparinux. Pregnant women as well as all other tested patients tolerated application of fondaparinux well without local or systemic side effects. We therefore recommend fondaparinux and danaparoid as alternative drugs in pregnant female patients with DTHR against LMWH.

In patients with an urgent indication for cardiac surgery, the selection of an adequate alternative is even more limited because of several reasons. In most of these cases, extra-corporeal circulation is needed during the operation. Until now, heparin sodium is the drug that can be controlled best during such an intervention because it has a short half-life and it can be antagonized by protamine sulfate [17].

Lepirudin is a potential alternative drug, but this drug has a long half-life, and the risk of bleeding is high because there is no antidote for this substance [17]. Furthermore, this drug may cause severe anaphylactic reactions, primarily in patients with repeated exposure to lepirudin and also in patients without previous contact [18-20]. It is remarkable that 2 of our patients developed DTHR after subcutaneous lepirudin administration, which is only rarely seen. One of these patients already had a positive reaction in intracutaneous testing, whereas the other one had none.

Bivalirudin, one of the newer synthetic direct thrombin inhibitors, is also a potential alternative for patients with an indication for heart surgery. The drug has already been used "on-" and "off-pump" in heart surgery in patients with heparin-induced thrombocytopenia or immunoglobulin E-mediated heparin allergy [21-23]. The advantages of this drug are its short half-life of about 25 minutes, and its predominant nonorgan elimination by proteolysis [22]. Furthermore, in contrast to hirudin, bivalirudin only binds transiently to the thrombin molecule. Bivalirudin has a low antigenic potential because it represents a small polypeptide; however, it shares a sequence of 11 amino acids with lepirudin [23]. Eichler and coworkers,[23] who investigated sera from 43 patients with antibodies against lepirudin could show that more than 50% of these sera were also reactive with bivalirudin. Therefore, one should be cautious of using bivalirudin in patients pretreated with lepirudin. In patients with a history of anaphylaxis after lepirudin exposure, bivalirudin should be avoided.

Argatroban is another synthetic thrombin inhibitor that inhibits free as well as fibrin- and clot-bound thrombin [24]. Theoretically, this drug is also an alternative in patients with heparin allergy and in need of cardiac surgery. Up to now, there is only limited experience with this drug in cardiac surgery [25]. Similar to other thrombin inhibitors, there is no antidote available. However, there are several reports of cardiac surgery performed successfully using argatroban as an anticoagulant [24,26,27]. All other drugs that are available as alternatives to UFH have to be considered as inappropriate at the present stage of knowledge.

In our patients, we tested neither bivalirudin nor argatroban because there is limited experience with these drugs and because heparin is ideal for the setting of cardiac surgery. In principle, bivalirudin as well as argatroban may be tested before heart surgery, but this should always depend on the recommendation of the treating hemostaseologists, anesthesiologists, and heart surgeons.

In patients with heart diseases investigated in this case series, the diagnostic procedure was always discussed interdisciplinary before allergologic testing. As in all cases, heparin sodium seemed to be the drug that can be controlled best under heart surgery; we performed an intravenous challenge with heparin sodium as previously described. The intravenous provocation was well tolerated in all cases. This observation is consistent with the results of Gaigl and coworkers [28] who performed intravenous heparin sodium provocation in 28 patients with a history of DTHR against subcutaneously injected heparin sodium. In their cohort, this challenge was well tolerated in all cases. The reason for intravenous tolerance despite development of DTHR after administration of the same drug subcutaneously is probably a phenomenon called *compartment allergy *based on a difference in antigen processing and presentation and preferential homing of selectively sensitized lymphocytes in the dermis [3]. Anyhow, hematogenous DTHR such as the development of the baboon syndrome cannot be excluded, but seems to be negligible in patients with an urgent indication of heart surgery. In emergency cases, intravenous application of heparin sodium may be performed even without prior allergologic testing, although development of maculopapular exanthemas or flare-ups cannot be excluded [9]. We also prefer intravenous provocation of heparin sodium in patients in need of cardiac surgery instead of using lepirudin, bivalirudin, or argatroban [28].

Until now, it is still unclear if patients with a history of DTHR have an increased risk of developing systemic complications such as thrombosis, skin necrosis, or HIT type II after reexposure to heparin. Harenberg and coworkers [29] investigated 9 patients with cutaneous heparin-induced allergic reactions after subcutaneous heparin application. Three (33.4%) of 9 patients showed pathological heparin-induced platelet activation (HIPA). Two of 3 HIPA-positive patients showed elevated heparin-induced immunoglobulin G titers and developed skin necrosis or heparin-induced thrombosis upon heparin administration, without decrease of platelet count. Aside from the report of Harenberg et al,[29] there is not much evidence for a coincidence of DTHR after subcutaneous heparin administration and the development of systemic complications or the production of heparin antibodies. Larger trials including allergologic testing as previously described and extensive laboratory investigation such as evaluation of heparin antibodies or HIPA are still missing. Under this aspect, potential systemic complications must be accepted, at least in patients with an urgent indication of cardiac surgery.

As allergic reactions after heparin administration may belong to different types of allergic reactions according to the Coombs and Gell classification, we recommend the following test procedures in patients with a history of heparin allergy: intracutaneous testing of UFH, LMWH, heparinoids, hirudins, and fondaparinux as previously described. Prick testing is not necessary in patients with DTHR and should only be performed in special situations, for example, in patients with a history of type I reactions after heparin administration. Patch testing can be omitted because of reduced sensitivity. Intracutaneous testing should be followed by subcutaneous challenge with drugs that had been negative in intracutaneous and patch testing. The repertory of drugs tested must be reduced in pregnant women and patients with an indication of cardiac surgery according to contraindications of several drugs in pregnancy and the actual recommendation of the responsible hemostaseologists, anesthesiologists, or heart surgeons. Up to now, there is no indication to test bivalirudin or argatroban routinely in patients with DTHR after heparin exposure. Biopsies from allergic skin reaction may help identify type II or III reactions.

## Note

The results of this clinical investigation have been presented in part at the "20. Mainzer Allergie-Workshop" of the German Society for Allergology and Clinical Immunology, March 7-8, 2008, in Mainz, Germany.
